# Identification of immune infiltration and cuproptosis-related subgroups in Crohn’s disease

**DOI:** 10.3389/fimmu.2022.1074271

**Published:** 2022-11-17

**Authors:** Yifan Yuan, Mingyue Fu, Na Li, Mei Ye

**Affiliations:** ^1^ Department of Gastroenterology, Zhongnan Hospital, Wuhan University, Wuhan, Hubei, China; ^2^ Hubei Clinical Centre and Key Laboratory of Intestinal and Colorectal Diseases, Zhongnan Hospital, Wuhan University, Wuhan, Hubei, China

**Keywords:** cuproptosis, Crohn’s disease, immune infiltration, differentially expressed genes, machine learning

## Abstract

**Background:**

Crohn’s disease (CD) is a type of heterogeneous, dysfunctional immune-mediated intestinal chronic and recurrent inflammation caused by a variety of etiologies. Cuproptosis is a newly discovered form of programmed cell death that seems to contribute to the advancement of a variety of illnesses. Consequently, the major purpose of our research was to examine the role of cuproptosis-related genes in CD.

**Methods:**

We obtained two CD datasets from the gene expression omnibus (GEO) database, and immune cell infiltration was created to investigate immune cell dysregulation in CD. Based on differentially expressed genes (DEGs) and the cuproptosis gene set, differentially expressed genes of cuproptosis (CuDEGs) were found. Then, candidate hub cuproptosis-associated genes were found using machine learning methods. Subsequently, using 437 CD samples, we explored two distinct subclusters based on hub cuproptosis-related genes. Gene Ontology (GO), Kyoto Encyclopedia of Genes and Genomes (KEGG) pathway enrichment, Gene set variation analysis (GSVA) and immune infiltration analysis studies were also used to assess the distinct roles of the subclusters.

**Results:**

Overall, 25 CuDEGs were identified, including ABCB6, BACE1, FDX1, GLS, LIAS, MT1M, PDHA1, etc. And most CuDEGs were expressed at lower levels in CD samples and were negatively related to immune cell infiltration. Through the machine learning algorithms, a seven gene cuproptosis-signature was identified and two cuproptosis-related subclusters were defined. Cluster-specific differentially expressed genes were found only in one cluster, and functional analysis revealed that they were involved in several immune response processes. And the results of GSVA showed positive significant enrichment in immune-related pathways in cluster A, while positive significant enrichment in metabolic pathways in cluster B. In addition, an immune infiltration study indicated substantial variation in immunity across different groups. Immunological scores were higher and immune infiltration was more prevalent in Cluster A.

**Conclusion:**

According to the current research, the cuproptosis phenomenon occurs in CD and is correlated with immune cell infiltration and metabolic activity. This information indicates that cuproptosis may promote CD progression by inducing immunological response and metabolic dysfunction. This research has opened new avenues for investigating the causes of CD and developing potential therapeutic targets for the disease.

## Introduction

Inflammatory bowel disease (IBD), a common inflammatory illness, has a complicated etiology that involves genetic and environmental triggers. These triggers include, but are not limited to, poor bacterial clearance, impaired mucosal barrier function, and ongoing dysregulation of immune responses to symbiotic gut flora (1, 2). IBD is subdivided into ulcerative colitis (UC) and Crohn’s disease (CD) based on several disease phenotypes (3). Additionally, CD is seen as a worldwide health issue, its incidence is accelerating in newly industrialized countries, and it has a high prevalence in Western countries (4). Thus, understanding the etiology and pathogenesis of CD may contribute to guiding clinical diagnosis and treatment, leading to improved clinical outcomes.

Traditionally, cells may die through programmed cell death, including pyroptosis, necroptosis, apoptosis, NETosis, or necrosis (5). But lately, cuproptosis, a novel mode of cell death, has been identified. Cuproptosis is a process caused by direct binding of copper ions to fatty acylated components of the tricarboxylic acid cycle in mitochondrial respiration and it causes fatty acylated proteins to stick together, iron-sulfur clusterin to be turned down, and eventually the cell to die (6, 7). The maintenance of copper ions (Cu^2+^) can act as cofactors for enzymes, and copper homeostasis is regulated mainly by mitochondria (8). Copper ionophores are tiny molecules bonded to copper that transport copper into cells and serve as valuable research tools for determining copper toxicity (9, 10). The mechanism by which copper ionophores induce cell death involves the accumulation of intracellular copper rather than the action of the small molecular chaperones themselves (6). Previous studies show that serum Cu^2+^ levels in IBD patients compared with healthy controls are controversial (11, 12). Epithelial mitochondrial dysfunction is related to the pathophysiology of CD (8, 13). In addition, genome-wide association studies found roughly 200 IBD risk loci (14), of which about 5% are functionally involved in maintaining mitochondrial health (15). In the gut, mitochondrial metabolism and function play critical roles in immune cell activation (16, 17). We may thus conclude that cuproptosis and the pathophysiology of CD are strongly connected. But the precise process is still a mystery.

To explore possible pathogenesis, we used the Gene Expression Omnibus (GEO) database to analyze the genes that are differently expressed between normal and CD samples. Then, we extracted differential genes and cuproptosis-related genes for intersection to find the differentially expressed cuproptosis-related genes (CuDEGs). In addition, multiple machine learning algorithms are applied to find the critical differential genes. We divided 437 CD patients into two groups that were connected to cuproptosis based on the seven hub CuDEGs expression patterns, and the variations in immune cells between the two groups were further analyzed. Finally, we consider the relationship between cuproptosis and immune infiltration to provide a novel angle of view to better understand the underlying molecular mechanism in CD pathogenesis.

## Materials and methods

### Acquisition of datasets and cuproptosis-related genes

We used the “GEOquery” R (18) program to get two raw datasets (GSE112366 (19) and GSE75214 (20)) from the GEO (https://www.ncbi.nlm.nih.gov/geo/) database (21), which included gene expression data for CD patients and controls. The GSE112366 dataset contains 362 CD samples and 26 normal samples, and GSE75214 contains 75 CD samples and 22 control samples. All the data was transformed into log2 form for subsequent analyses. The Bioconductor “sva” R software was used to get rid of the batch effects and make the unified GEO dataset (22).

The cuproptosis-related genes in the Molecular Signature Database (MsigDB) v7.0 database (23) (http://www.gsea-msigdb.org/gsea/msigdb/) were combined with gene sets relevant to cuproptosis from a prior study (6). After we got rid of the duplicates, we found 52 genes that are involved in cuproptosis.

### Identification of differentially expressed genes associated with CD and cuproptosis

We utilized the R package “limma” (24) to do the differential gene analysis, and the threshold of P<0.05 was chosen as the threshold to identify differentially expressed genes (DEGs) between the illness and health samples in the combined dataset. Differential gene expression data was shown using volcano plots and a heatmap. Gene Ontology (GO) enrichment analysis and Kyoto Encyclopedia of Genes and Genomes (KEGG) pathway analysis were also performed in R using the “clusterProfiler” package (25) to further investigate the biological roles of DEGs.

### Evaluating the immune cell infiltration

Immunology cells, inflammatory cells, mesenchymal tissues, fibroblasts, and various cytokines and chemokines serve as the immunological microenvironment composition. Analysis of immune cell infiltration has a crucial driving function in understanding disease progression and therapeutic response. An extension of the GSEA methodology, the single sample gene set enrichment analysis (ssGSEA), was built using 23 immune gene sets. The immunologic features of all samples were evaluated using the ssGSEA technique using the “GSVA” R package (26).

### Machine learning

By intersecting DEGs and cuproptosis-associated genes, the genes that are differentially expressed and connected to cuproptosis were identified as CuDEGs. Three machine-learning techniques were used to further filter the list of potential genes for CD diagnosis. The least absolute shrinkage and selection operator (LASSO) is a regression approach used for regularization to increase predicted accuracy and model comprehensibility and choose variations (27). Support vector machine (SVM) is a powerful method whose goal is to establish a threshold between two classes, allowing for label prediction based on a single or several feature vectors (28). To predict continuous variables and provide predictions with little to no apparent fluctuation, random forest (RF) is a suitable technique with the advantages of no constraints on variable conditions and greater accuracy, sensitivity, and specificity (29). The LASSO regression, SVM, and RF analyses were carried out using the R packages “glmnet” (30), “kernlab” (31) and “randomForest” (32). The intersection genes of them were considered as hub cuproptosis genes in CD diagnosis. And the “circlize” R package (33) was used to illustrate the interaction between the hub cuproptosis genes. The receiver operating characteristic (ROC) analysis was performed using the “pROC” R package (34) to further evaluate the prediction model’s capacity to discriminate CD from non-CD controls.

### Subclusters analysis with seven cuproptosis-related genes

Using the “ConsensusClusterPlus” R package (35) and the mRNA expression of seven Cuproptosis-related genes as input information, an unsupervised hierarchical clustering analysis was performed on the 437 CD samples. When we looked at the subclusters using a PCA plot, we could see the geometrical distance between them. GSVA (26) was performed to clearly state the functional distinctions between the Cuproptosis subclusters found *via* previous cluster analysis. The “h.all.v7.5.1.symbols”, “c2.cp.kegg.v7.5.1.symbols” and “c2.cp.reactome.v7.5.1.symbols” files were downloaded for GSVA analysis from the MSigDB online database. After that, a heatmap was utilized to show how the two subclusters of genes involved in cuproptosis were distinct from one another in terms of the activity of their respective pathways. With a |log2 fold change (FC)| > 0.5 and an adj. p < 0.05 being considered statistically significant, the DEGs were identified between the two Cuproptosis-related subclusters and were shown using a volcano plot. Following that, GO and KEGG enrichment analyses were performed to depict their biological functions *via* the “clusterProfiler” package (25).

### Statistical analysis

Data analysis and statistical analyses were accomplished by R 4.1.2. Using the Wilcox test, the statistical difference between the two groups was assessed. A Spearman correlation was used to analyze the relationship between the expression levels of genes associated with hub cuproptosis and immune cells. A p-value of 0.05 is statistically significant.

## Results

### Merging GEO data and identification of DEGs

**Figure 1:**
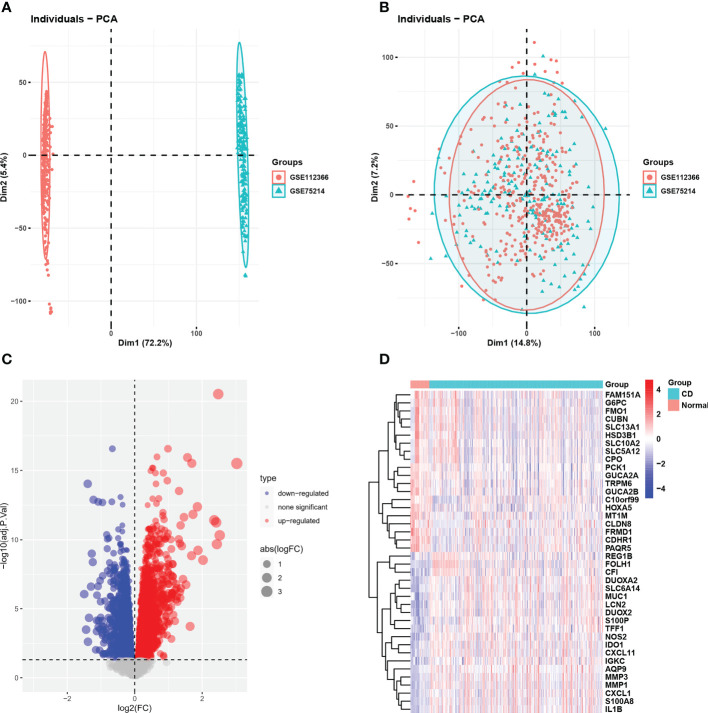
GEO dataset combination and differentially expressed genes. **(A, B)** PCA showed the batch effect between integrated datasets before and after de-batching. **(C)** The volcano plot of CD-related DEGs. **(D)** Clustered heatmap of CD-related DEG expression levels. GEO, gene expression omnibus; PCA, principal component analysis; CD, Crohn’s disease; DEGs, differentially expressed genes.

The integrated dataset consists of 437 CD samples and 46 control samples and was obtained by removing the batch effects from the GEO dataset (**Figures 1A, B**). We identified a total of 5865 DEGs using the Limma method, and the volcano plot and heatmap of DEGs are shown in **Figures 1C, D**.

### Functional and pathway enrichment analysis of DEGs

**Figure 2:**
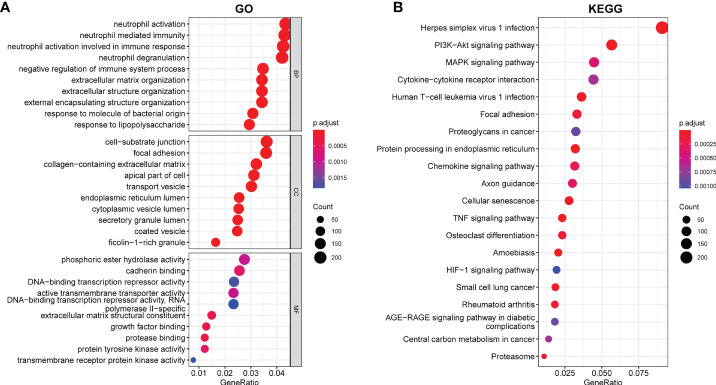
Enriched items in GO and KEGG analyses using DEGs. **(A)** Enriched items in GO analysis. **(B)** Enriched items in KEGG pathway analysis. GO, Gene Ontology; BP, Biological process; CC, Cellular component; MF, Molecular function; KEGG, Kyoto Encyclopedia of Genes and Genomes.

We conducted a GO and KEGG enrichment pathway analysis in R to investigate the possible role of these genes. The GO enrichment analysis revealed enrichment of neutrophil mediated immunity, negative regulation of the immune system process, neutrophil activation, response to molecule of bacterial origin, and response to lipopolysaccharide. Moreover, we also demonstrated enrichment of cell-substrate junction, focal adhesion, collagen-containing extracellular matrix, ficolin-1-rich granule, and transport vesicle. Furthermore, in molecular function, growth factor binding, enrichment of DNA-binding transcription repressor activity, transmembrane receptor protein kinase activity, and protein tyrosine kinase activity were discovered (**Figure 2A**). According to the KEGG analysis, DEGs were found to be enriched in the following items according to the KEGG analysis: PI3-Akt signaling pathway, MAPK signaling pathway, chemokine signaling pathway, TNF signaling pathway, and HIF-1 signaling pathway (**Figure 2B**).

### Evaluation of immune cell infiltration

**Figure 3:**
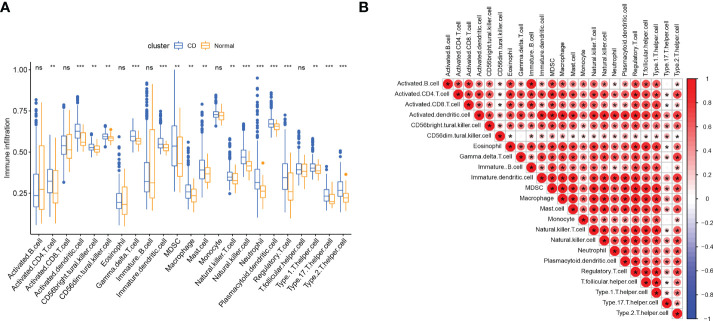
Immune cell infiltration in patients with CD. **(A)** Correlation matrix of all 23 types of immune cell subtype compositions. **(B)** Comparison of 23 immune cell subtypes between patients in CD and controls; The size and color of the circle represent the Pearson correlation coefficients. ns, no significance; * represents p < 0.05, ** represents p < 0.01; *** represents p < 0.001.

Since we found that immune-related genes might affect CD pathogenesis, we did an immune cell infiltration investigation to shed further light on the immunological regulation of CD. The boxplot showed that CD patients had a higher proportion of activated CD4 T cells, activated dendritic cell (DC), CD56bright natural killer (NK) cells, CD56dim NK cells, gamma delta T cells, myeloid-derived suppressor cells (MDSCs), macrophage, mast cell, NK cell, Neutrophil, plasmacytoid Dendritic Cells (pDCs), regulatory T cells (Tregs), T helper 1 (Th1) cells, T helper type 17 (Th17) cells, and T helper 2 (Th2) cells, while the activated B cell and immature B cell did not show a significant difference (**Figure 3A**). The association between 23 categories of immune cells demonstrated that most infiltrating immune cells were closely connected, except for CD56dim NK cells or Th17cells (**Figure 3B**). Diverse types of immune cells were infiltrated uniquely in CD patients, which might serve as a possible therapeutic target for CD.

### Identification of the cuproptosis-signature via machine learning

**Figure 4:**
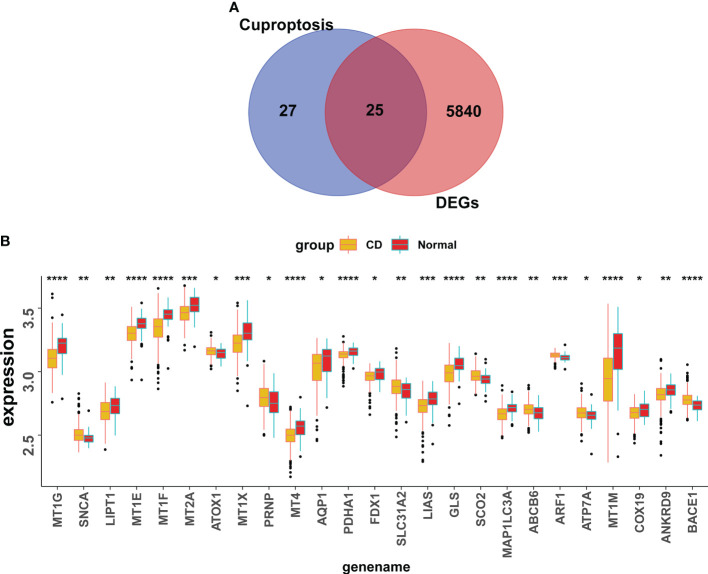
Screening of the CuDEGs in CD. **(A)** The overlap of genes between DEGs and cuproptosis-related genes. **(B)** Overall expression histogram of CuDEGs in CD patients. CuDEGs; differentially expressed cuproptosis-related genes. * represents p < 0.05, ** represents p < 0.01; *** represents p < 0.001; ****P<0.0001.

**Figure 5:**
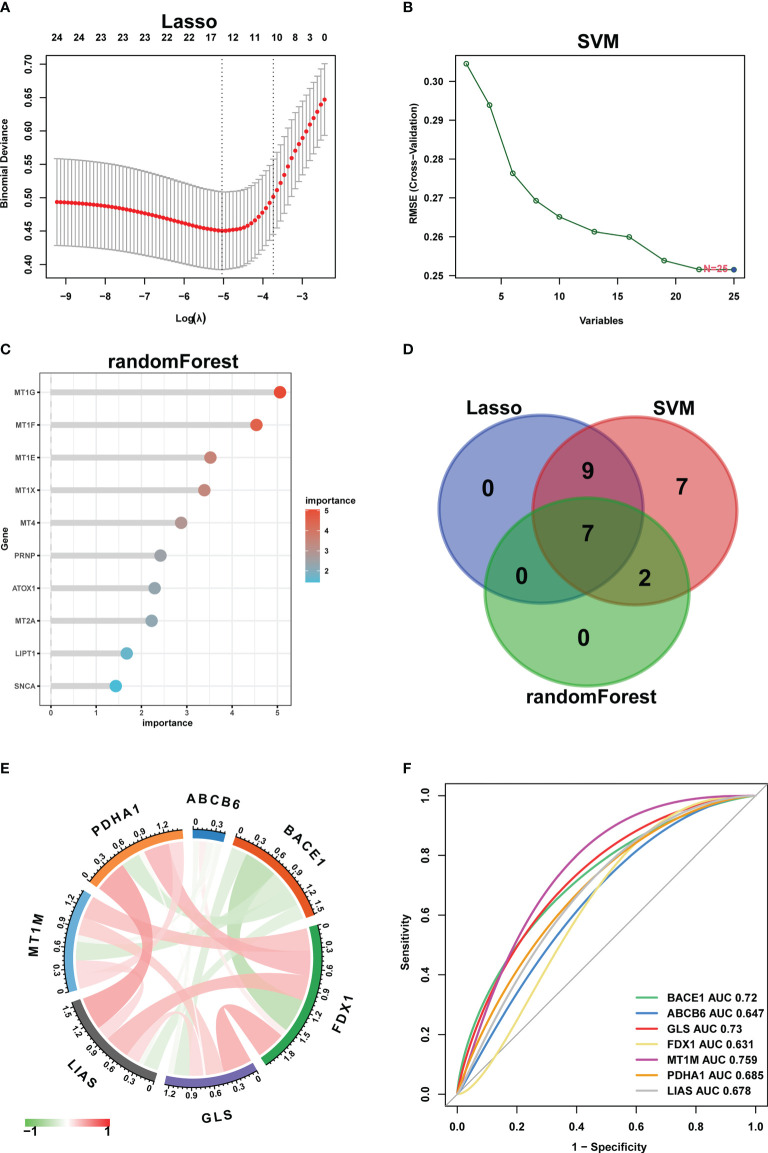
Machine learning in the identification of cuproptosis-signature. **(A–C)** Construction of cuproptosis-signatures using LASSO regression, SVM, and RF algorithm. **(D)** The Venn diagram shows the overlap of candidate genes between the above three algorithms. **(E)** Circos plot displaying the relationship between the overlapping cuproptosis genes. **(F)** ROC curve of cuproptosis-signatures in CD diagnosis. LASSO, least absolute shrinkage and selection operator; SVM, support vector machine; RF, random forest; ROC, receiver operating characteristic.

25 CuDEGs were screened after the intersection of the 5765 DEGs and 52 cuproptosis-related genes (**Figure 4A**). And the overall expression of CuDEGs between the CD samples and the normal samples is shown in **Figure 4B**. What’s more, most CuDEGs were expressed at lower levels. After obtaining CuDEGs, the LASSO regression technique, SVM, and random forest algorithms were utilized to screen potential genes for the construction of the cuproptosis-signature (**Figures 5A–C**). Finally, a 7-gene cuproptosis-signature, including BACE1, ABCB6, GLS, FDX1, MT1M, PDHA1, and LIAS, was identified (**Figure 5D**). As shown in **Figure 5E**, most cuproptosis-signature genes, except ABCB6 and BACE1, are highly correlated with each other (**Figure 5E**). To predict CD, the ROC curves of seven gene signatures were analyzed. Notably, MT1TM had the highest AUC among the seven hub genes, with a value of 0.759. Other AUC values for BACE1, ABCB6, GLS, FDX1, PDHA1, and LIAS were 0.72, 0.649, 0.73, 0.631, 0.685, and 0.678, respectively (**Figure 5F**). These results indicated that all seven gene signatures had excellent diagnostic values.

### The relationship of cuproptosis-related genes with immune cells and GSEA

**Figure 6:**
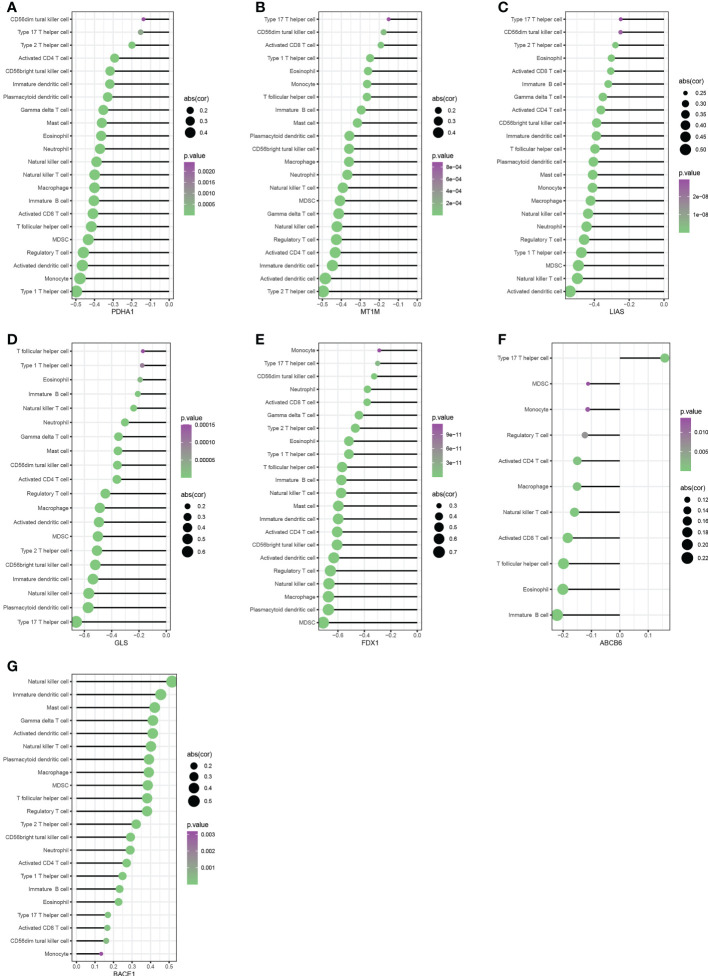
Correlation between immune infiltrating cells with PDH1, MT1M, LIAS, GLS, FDX1,ABCB6 and BACE1 **(A–G)**.

**Figure 7:**
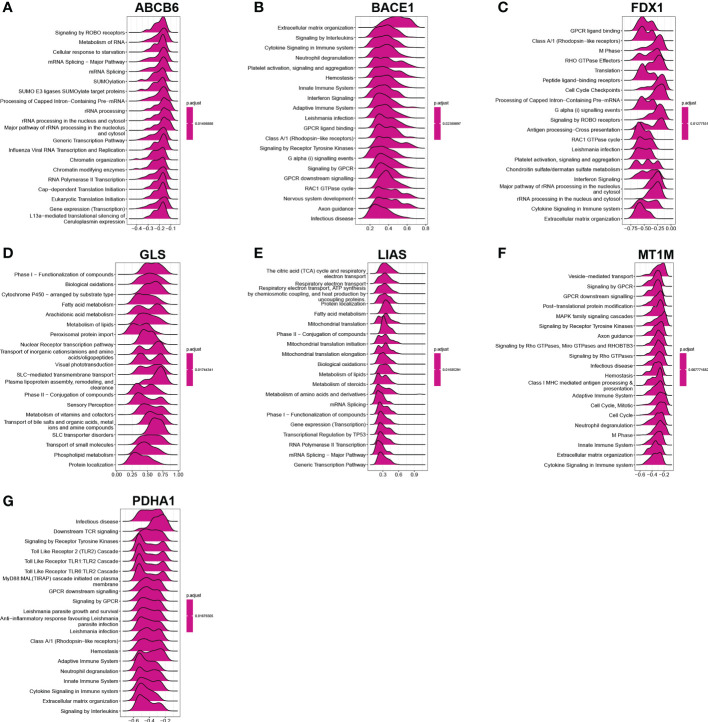
GSEA investigation of ABCB6, BACE1, FDX1, GLS, LIAS, MT1M, and PDHA1 **(A–G)**.

We performed Spearman correlation analysis to see whether these diagnostic genes were associated with immune cell infiltration in order to better understand the function of these hub cuproptosis-related genes in immune infiltration. Five diagnostic genes, including PDH1, MT1M, LIAS, GLS, and FDX1, exhibited a strongly negative relationship with the infiltration of most immune cells, according to correlation analysis (**Figures 6A–E**). And ABCB6 had a significantly negative relationship with 10 kinds of immune cells (**Figure 6F**). Interestingly, BACE1 is the only gene that had a significantly positive relationship with the infiltration of almost all the immune cells(**Figure 6G**). As shown in **Figures 7A–G**, the ABCB6, BACE1, FDX1, GLS, LIAS, MT1M, and PDHA1 were strongly enriched in metabolic and immune-related pathways.

### Consensus clustering analysis of cuproptosis gene clusters

**Figure 8:**
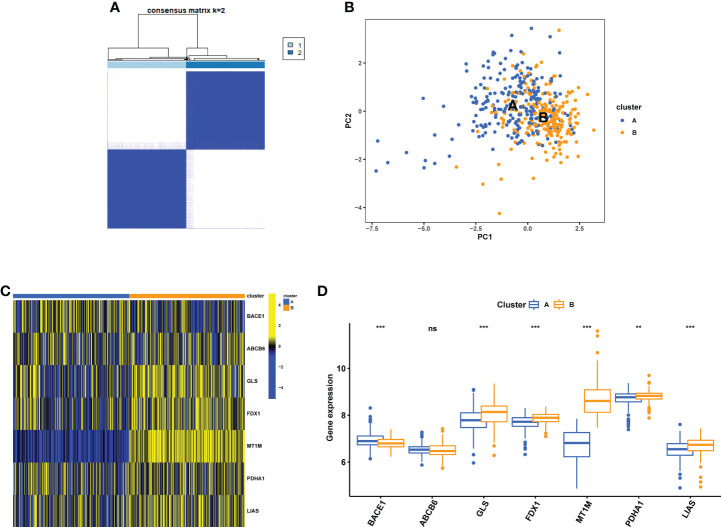
Identification of cuproptosis subtypes in CD. **(A)** Subclusters were performed with differential genes. **(B)** PCA diagram showing the distribution of different subclusters. Heatmap **(C)** and boxplot **(D)** show differential expression of CuDEGs between subtypes. ns, no significance; ** represents p < 0.01; *** represents p < 0.001.

Consensus clustering analysis was performed using the R software’s “Consensus Cluster Plus” package. The 7 cuproptosis-related gene expressions were used to determine that k = 2 provided the most stable grouping (**Figure 8A**). Since then, the 437 CD samples that were obtained from the GEO database have been split into two distinct categories in consensus clustering analysis, i.e., cluster A (low expression group, n = 219) and cluster B (high expression group, n = 218). As shown by the PCA plot, gene expression patterns were distinct between the clusters (**Figure 8B**). The expression level of the CuDEGs in the two subtypes was visualized through the heatmap and boxplot (**Figures 8C, D**). Likely, most cuproptosis-related genes, including GLS, FDX1, MT1M, PDHA1, and LIAS, have greater expression levels in cluster B than in cluster A, although ABCB6 and BACE1 do not.

### GSVA of biological pathways between subclusters of cuproptosis

**Figure 9:**
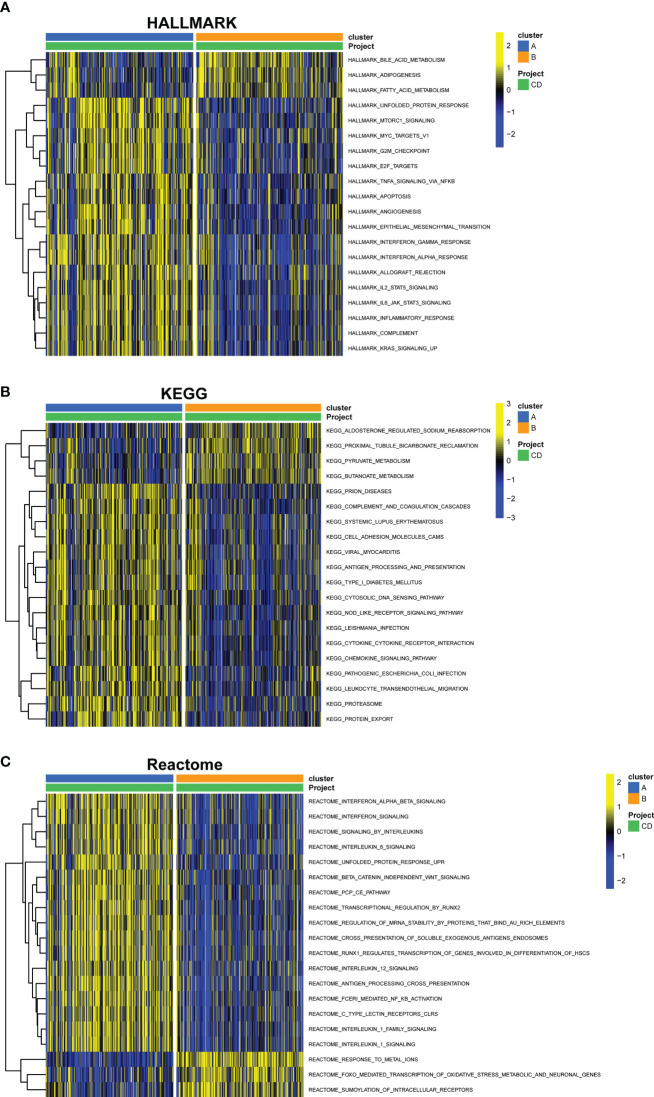
GSVA of key pathways between cuproptosis subtypes. **(A)** Enriched pathways based on the HALLMARK pathway. **(B)** Enriched pathways based on the KEGG pathway. **(C)** Enriched pathways based on the Reatcome pathway. GSVA, gene set variation analysis.

Through GSVA analysis, several pathways with differential expression were enriched, and they were shown in a heatmap. Compared with cluster B, Hallmark activities of mTORC1 signaling, apoptosis, interferon-interferon-α, TNF-α signaling, IL2-STAT5 signaling, and IL6-JAK-STAT3 signaling were higher in cluster A, while bile acid metabolism, adipogenesis, and fatty acid metabolism were lower (**Figure 9A**). In cluster A, the expression of KEGG pathways linked with pyruvate and butanoate metabolism was dramatically reduced, whereas the expression of NOD-like receptor signaling, cytokine-cytokine receptor interaction, chemokine signaling, and leukocyte transendothelial migration associated pathways were significantly higher (**Figure 9B**). Based on the Reatcome-based pathway, the results of GSVA showed significant enrichment in interferon signaling, interleukin6 signaling, interleukin12 signaling, and antigen processing cross presentation related pathways in cluster A. In contrast, response meta ions FOXO mediated transcription of oxidative stress metabolic and neuronal genes and SUMOylation of intracellular receptors were enriched in cluster B (**Figure 9C**).

### Functional distinctions between the two cuproptosis subclusters

**Figure 10:**
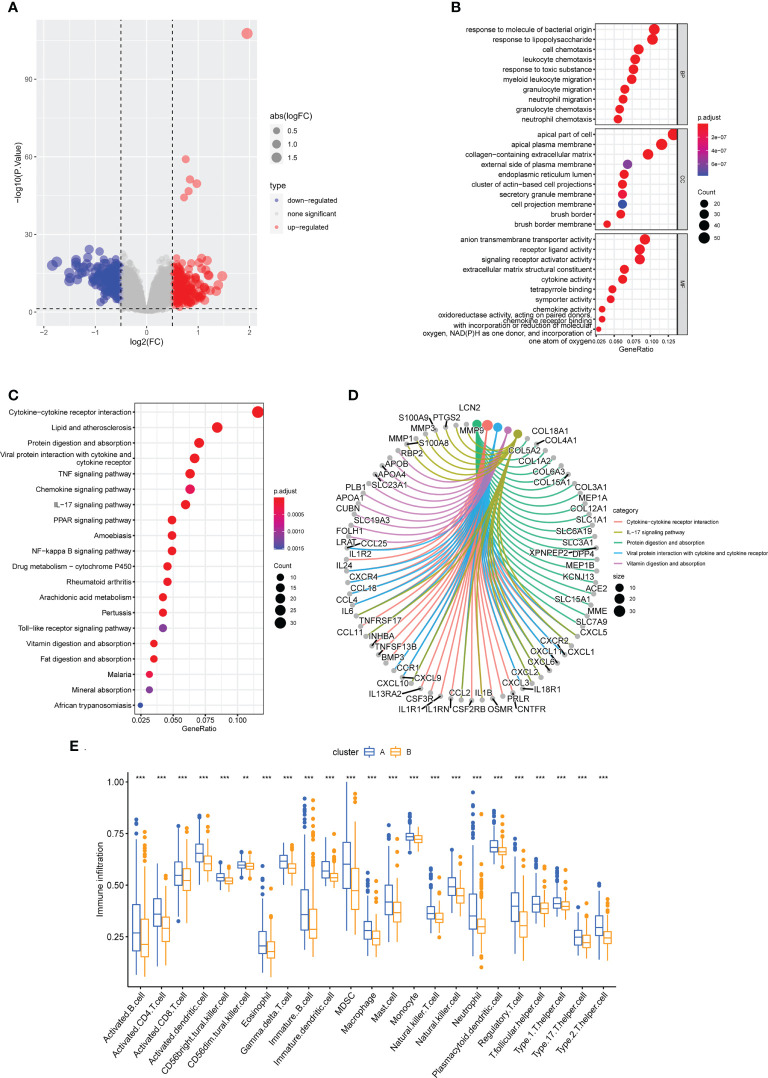
Functional enrichment analysis and immune cell infiltration between cuproptosis subtypes. **(A)** The volcano plot of DEGs. **(B)** Enriched items in GO analysis. **(C, D)** Enriched items in KEGG pathway analysis. **(E)** Correlation matrix of all 23 immune cell subtype compositions. ** represents p < 0.01; *** represents p < 0.001.

To learn more about the functional differences between the two subclusters, a differential expression analysis was conducted. A total of 440 DEGs, comprising 205 upregulated and 235 downregulated DEGs, were obtained. The distribution of DEGs between them was shown on the volcano plot (**Figure 10A**). The 440 DEGs were then subjected to GO and KEGG enrichment analysis to further understand the probable molecular processes and functions. Genes were enriched in cell chemotaxis, leukocyte chemotaxis, myeloid leukocyte migration, granulocyte migration, and neutrophil migration, as indicated by GO analysis (BP); apical plasma membrane, external side of plasma membrane secretory granule membrane, cell projection membrane and brush border membrane (CC); signaling receptor activator activity, cytokine activity, chemokine activity, and oxidoreductase activity, acting on paired donors, with incorporation or reduction of molecular oxygen (MF) (**Figure 10B**). What’s more, the KEGG enrichment analysis found that these genes were mostly enriched in the Cytokine−cytokine receptor interaction, IL−17 signaling pathway, Protein digestion and absorption, Viral protein interaction with cytokine and cytokine receptor, Vitamin digestion and absorption, and some other immune system related pathways (**Figures 10C, D**). Intriguingly, consistent with the findings of the GSVA analysis, which indicated that the overall immune infiltration was greater in cluster A, cluster A patients were found to have considerably higher levels of all 23 immune cells in the ssGSEA. (**Figure 10E**).

## Discussion

CD remains hard to cure because of its heterogeneity. The molecular mechanisms of this disease are still complex and not completely understood. CD treatment has changed with the advancement of tumor necrosis factor-inhibitors beyond the scope of conventional treatment. Many patients may not react to or are unable to maintain therapy with these medications, which have a variety of adverse effects, despite their long-term success (36). Therefore, it has become vital to create novel medications that target specific pathways in the pathophysiology of CD. So, our study links cuproptosis to the pathogenesis of CD, identifies possible key genes through bioinformatics analysis, and explores potential therapeutic targets.

We used the GEO database to investigate the gene expression levels of healthy controls and CD patients, and we finally identified 5865 DEGs. The GO enrichment analysis revealed enrichment of neutrophil activation, neutrophil mediated immunity, and negative regulation of the immune system process. Neutrophils play an important role in IBD gut inflammation and neutrophil activation correlates with disease activity in IBD, according to several previous studies (37–41). In our study, DEGs were found to be involved in the PI3-Akt signaling pathway, MAPK signaling pathway, and TNF signaling pathway according to the KEGG analysis. Consistent with the previous studies, the PI3-Akt signaling pathway, the MAPK signaling pathway, and the TNF signaling pathway have been discovered to be related to intestinal cell death (41–43). This partly explains the death of intestinal epithelial cells.

Innate and adaptive immune cells in CD are triggered by DC, NK, and T cell subsets. T cells that generate pro-inflammatory cytokines may maintain inflammation and tissue damage, whereas T cells that produce anti-inflammatory cytokines enhance barrier function and inflammation resolution. Th17/Tregs imbalances may also induce CD, according to previous investigations (44). However, it is not clearly established whether plasma cells or B lymphocytes play a key role in the pathogenesis of CD. Therefore, so far, B cell-targeting is not part of the therapeutic armamentarium in CD (45).The abundance of immune cells showed that CD patients exhibited higher infiltration levels of activated CD4 T cells, activated dendritic cells, CD56bright NK cells, CD56dim NK cells, gamma delta T cells, immature B cells, and so on, which is consistent with previous research (46). This further demonstrates the importance of immunity in the development of CD.

Several studies have shown that increased apoptotic epithelial cell death in patients with IBD (47), and cuproptosis is a newly discovered way of programmed cell death (6, 7). However, further research is needed into cuproptosis’ regulatory involvement in different disorders and its underlying processes. As a result, we attempted to better comprehend the function of cuproptosis-related genes in CD phenotyping. For the first time, we compared the expression patterns of CuDEGs regulators in colon tissues from healthy individuals and CD patients. Patients with CD had decreased levels of the dysregulated CuDEGs compared to healthy controls, indicating an important function for CuDEGs in the development of CD. Next, three machine learning classifiers were used to select seven hub genes (BACE1, ABCB6, GLS, FDX1, MT1M, PDHA1, and LIAS). For further elucidation of the connection between cuproptosis regulators and CD, we then determined the correlation among hub CuDEGs. We discovered that CuDEGs interactions in CD patients provided strong evidence of synergistic or antagonistic effects. BACE1 encodes a member of the peptidase A1 family of aspartic proteases, and both BACE1 expression and activity are elevated in the aging brain and Alzheimer’s disease (AD) (48–51), as well as in response to oxidative stress and inflammation (52, 53). ABCB6 can modulate the survival of megakaryocyte progenitors during oxidative stress (54). GLS is an essential molecule for Th17 cell production, and GLS deficiency protects against inflammatory bowel disease (55). FDX1 expression in colon adenocarcinoma (COAD) is positively associated with “quiescence” and “inflammation” but negatively correlated with “invasion”, and the expression of FDX1 is positively correlated with infiltration levels of CD8+ T cells, NK cells, and neutrophils (56). It is reported that up-regulation of MT1M can inhibit RGC cell apoptosis and inflammation (57). PDHA1 can promote NLRP3 inflammasome activation (58). LIAS encodes the protein of the biotin and lipoic acid synthetases family and this iron-sulfur enzyme is located in the mitochondrion (59). Decreased LIAS expression is associated with diminished hepatic alpha-lipoic acid and tissue oxidative stress (60). However, the relevance of these genes in CD has not been documented and further research is required.

Based on the seven hub genes, we utilized unsupervised cluster analysis to identify two distinct cuproptosis-related clusters. And in the subcluster function analyses, GSVA analysis indicated that cluster A exhibited elevated, relatively higher levels of immune-related pathways and lower metabolic related pathways, and with lower expression of cuproptosis-related genes, higher levels of immune infiltration. In addition, in GO analysis, DEGs were primarily enriched in cell chemotaxis, apical plasma membrane and signaling receptor activator activity in cluster A, and in KEGG analysis, these genes were primarily enriched in the cytokine−cytokine receptor interaction, IL−17 signaling pathway, and protein digestion. It is well known that CD is an immune-related disease. Th17 activation contributes to the formation of granulomas (61). Some antigens made by bacteria can cause CD4+ T cells to change into targeted cytotoxic T lymphocytes. These cells then release interleukin-17 to stimulate Th17 cells to make transforming growth factor and interferon-alpha, which can cause persistent inflammation and fibrosis (62).

This study has several limitations as well. First, our results need to be further demonstrated because of the lack of confirmation from experiments and clinical trials. All the above results are based on comprehensive bioinformatics analysis. What’s more, our data comes from a public database, and we lack raw sequencing data, so there may be a selection bias. Well-designed prospective studies are necessary to further verify our results. Besides, since the sample size was still relatively small, we needed a greater number of CD subjects to prove our results. Further studies are necessary to clarify the underlying mechanisms. In addition, the GSE112366 database used in this investigation contains some samples after treatment with Ustekinumab. Whether this therapy has any impact on gene expression is currently unknown. Lastly, the possibility that GSE112366 contains samples from the same person may present a risk to the validity of this study’s findings.

## Conclusion

Through a series of bioinformatic techniques, our work uncovered the association between cuproptosis-related genes and infiltrated immune cells, as well as the great heterogeneity of immunological responses among CD patients with distinct cuproptosis subclusters. A seven-gene-based cuproptosis-signature was selected as the optimal machine learning model, which can accurately assess CD subtypes and the diagnosis of CD patients. Our findings provide light on the involvement of cuproptosis in the progress of CD for the first time, and provided new insight into the underlying pathogenic processes and potential CD treatment strategies.

## Data availability statement

The datasets presented in this study can be found in online repositories. The names of the repository/repositories and accession number(s) can be found in the article/supplementary material.

## Author contributions

YY designed the study, collected the original data and finished the analysis. YY and MF drafted the initial manuscript. NL helped revise the manuscript. MY provided the funding and supervised the study. The final manuscript was read and approved by all authors. All authors contributed to the article and approved the submitted version.

## Funding

This research was supported by the National Natural Science Foundation of China (No. 81870391) and the Key Project of Medical Science and Technology Innovation Platform Construction Support of Zhongnan Hospital of Wuhan University (PTXM2022007).

## Conflict of interest

The authors declare that the research was conducted in the absence of any commercial or financial relationships that could be construed as a potential conflict of interest.

## Publisher’s note

All claims expressed in this article are solely those of the authors and do not necessarily represent those of their affiliated organizations, or those of the publisher, the editors and the reviewers. Any product that may be evaluated in this article, or claim that may be made by its manufacturer, is not guaranteed or endorsed by the publisher.
